# NeQuick-G and Android Devices: A Compromise between Computational Burden and Accuracy

**DOI:** 10.3390/s20205908

**Published:** 2020-10-19

**Authors:** Ciro Gioia, Daniele Borio

**Affiliations:** Joint Research Centre of the European Commission, 21027 Ispra, Italy; daniele.borio@ec.europa.eu

**Keywords:** NeQuick-G, Android, computational load, ionosphere

## Abstract

Ionospheric delay is one of the largest errors affecting Global Navigation Satellite System (GNSS) positioning in open-sky conditions, and different methods are currently available for mitigating ionospheric effects including dual-frequency measurements and corrections from augmentation systems. For single-frequency standalone receivers, the most widely used approach to correct ionospheric delays is to rely on a model. In this respect, Klobuchar and NeQuick-G Ionospheric Correction Algorithms (ICAs) are the approaches adopted by GPS and Galileo, respectively. While the latter outperforms the Klobuchar model, it requires a significantly higher computational load, which can limit its exploitation in some market segments such as smartphones. In order to foster adoption of the NeQuick-G model in this type of device, a smart application of NeQuick-G is proposed. The solution relies on the assumption that ionospheric delays are practically constant over short time intervals. Thus, the update rate of the ionospheric correction computation can be significantly reduced. This solution was implemented, tested, and evaluated using real data collected with a static smartphone in an ad hoc set-up. The impact of reducing the ionospheric correction update rate has been evaluated in terms of processing time, of ionospheric correction deviations and in the Ranging Error (RE) and position domains. The analysis shows that a significant reduction of the processing time can be obtained with negligible degradation of the navigation solution.

## 1. Introduction

The performance of Global Navigation Satellite System (GNSS)-based navigation is influenced by the errors present in the measurements used for Position, Velocity and Time (PVT) computation. GNSS observables are affected by different types of errors, specifically satellite clock related errors, relativistic effects, tropospheric and ionospheric delays, and multipath- and receiver-related errors. These errors are corrected/mitigated using models [1,2]. In open-sky conditions with good-quality signals, ionospheric delay is the largest error source, which is difficult to model accurately. Ionospheric errors can vary from a few meters to many tens of meters [3].

Different techniques can be adopted for mitigating the ionospheric effect, depending on the type of device used (single- or multi-frequency) and on the information available. The most effective method for mitigating the ionospheric effect is the use of multi-frequency signals: the linear combination of observables (i.e., iono-free combination) from two different frequencies allows the reduction of the ionospheric error up to 99.9% [4,5]. While the use of dual-frequency measurements has been mainly limited by the increased cost of multi-frequency devices, low-cost multi-frequency receivers have been recently introduced. Multi-frequency devices cover a large part of the professional receiver market segment with limited penetration in the mass-market sector. In the smartphone market, the first mobile phone equipped with a dual-frequency GNSS chip-set was introduced by Xiaomi in 2018 [6]. Although, several manufactures are currently producing dual-frequency devices, the adoption of a dual-frequency GNSS receiver in a smartphone is still an open discussion.

The quality of GNSS measurements obtainable from Android smartphones is strongly affected by the characteristics of the GNSS antenna and of the surrounding electronic elements. Mobile phones include different sensors, electronic components, and antennas (for WiFi, Bluetooth, 4G, etc.); interference between different hardware elements can degrade the performance of the integrated GNSS receiver [7]. Currently, out of the 424 Galileo-capable smartphones considered by the European GNSS Agency (GSA) market study [8], only 25% of them are equipped with a dual-frequency GNSS chip-set. Thus, 75% of the smartphones still rely on single-frequency measurements without the possibility to form iono-free combinations.

A first attempt to monitor ionosphere using Android devices is illustrated in [9]. A dual-frequency Precise Point Positioning (PPP) solution using a Xiaomi Mi8 device was demonstrated in [6]. The result shows that, for the dual-frequency solution, the time required for the three-dimensional positioning errors to converge to 1 m was about 102 min, while in the single-frequency case it was about 100 min. Non-specific advantages were demonstrated using dual-frequency measurements in [10], where the authors compared the performance of a dual-frequency smartphone with respect to a dual-frequency geodetic receiver. From the analysis, it emerged that in single-frequency mode, the two devices had a similar behavior. A standard deviation increase of about 5 m was observed when the comparison was performed using dual-frequency combinations. In [11], the authors explored the limitations of PPP implemented using smartphones. The paper focuses on the smartphone tracking performance, usability of smartphone phase data, and the benefits of Galileo observations. Although the authors identified the effects of the atmospheric delays as a major topic, they left the analysis for future works.

Single-frequency receivers correct the ionospheric error using models. Different methods can be adopted depending on the data available, including Global Ionospheric Map (GIM) [12], Klobuchar model [13], NeQuick-G [14], BDS-Klobuchar [15], Neustrelitz Total Electron Content Broadcast (NTCM-BC) [16], Satellite Based Augumentation System (SBAS) corrections [17,18]. Stand-alone single-frequency receivers adopt parametric models exploiting the parameters within the GNSS navigation message; the Klobuchar model and its modified version are quite simple to implement and are able to correct up to 50% of the ionospheric error.

Some advantages have been demonstrated when using the NeQuick-G model, which is able to correct up to 70% of the ionospheric error [14]. The performance of the different ionospheric models has been compared in several works. The assessment and comparison of broadcast ionospheric models are investigated in [19]. The improvements brought by NeQuick-G with respect to the Klobuchar models in the range and position domains were presented in [20]. The analysis was performed for different stations around the globe. The performance of the two models was evaluated in [21] for the Brazilian region. The impact of the ionospheric model in a PPP solution was evaluated in [22].

The main limitation of the NeQuick-G model is its computational load [23,24]. This aspect is of foremost importance for smartphones, which have limited computational capabilities. In order to foster the adoption of NeQuick-G in such types of devices, a smart application of the model is proposed. The approach exploits the assumption that the ionospheric error can be considered constant for short time intervals; hence, the update rate of the NeQuick-G corrections can be reduced without compromising the receiver performance. This smart application of the NeQuick-G model allows a significant reduction of the computational load. The approach developed has been tested using live data collected using a static smartphone. The impact of the proposed approach has been assessed in three domains: at the ionospheric correction, the Ranging Error (RE), and the position estimation level. The RE is computed considering all the corrections that can be applied in Single Point Positioning (SPP) mode. A detailed description on the computation of RE is provided in Section 2.2. For each domain, performance indicators were considered: the analysis shows that update intervals up to 30 s can be adopted with negligible performance degradations, as shown in Section 5. This reduction of the update rate provides a significant performance improvement in terms of processing time.

The proposed approach is not limited to smartphones and can be used in Internet of Things (IoT) applications, for which GNSS provides highly accurate and ubiquitous position and time information but requires relatively high energy consumption; this can be a limitation in the adoption of GNSS-based navigation for battery-powered IoT devices [25]. In these applications, a meter-level accuracy is required, and the proposed approach can help achieving this requirement while further reducing power consumption [26].

The remainder of the paper is organized as follows: a brief description of the Single Point Positioning (SPP) navigation algorithm is provided in Section 2. The NeQuick-G model and the proposed approach are detailed in Section 3, whereas the experimental set-up used for the data collection is illustrated in Section 4. Experimental results are discussed in Section 5, and finally, conclusions are drawn in Section 6.

## 2. Navigation Solution

In this section, the overall approach adopted for the evaluation of the navigation solution, including the computation of ionospheric corrections, is briefly introduced along with the definition of RE, one of the metrics adopted for the performance analysis.

### 2.1. Navigation Estimation

The position solution considered in this paper is based on a SPP approach, which evaluates the user position from the pseudoranges provided by the smartphone. Pseudoranges can be modeled as

(1) $$\rho =d+cdt_{s}-cdt_{r}+e_{rel}+e_{sag}+d_{Iono}+d_{Tropo}+\epsilon_{\rho }$$

where *d* is the distance between the satellite and receiver, $cdt_{s}$ is the satellite clock error, $cdt_{r}$ is the receiver clock error, $e_{rel}$ is the relativistic effect, and $e_{sag}$ is the error due to the Sagnac effect. $d_{Iono}$ and $d_{Tropo}$ are the ionospheric and tropospheric delays, respectively; finally, $\epsilon_{\rho }$ contains the errors due to multipath, receiver noise, and residual un-modelled effects [5].

In order to compute the user position, the receiver needs to apply different corrections that partially compensate for the errors in Equation (1). In particular, the corrected pseudorange, $\rho_{c}$, is obtained as

(2) $$\rho_{c}=\rho +satClock+Sag-I-T$$

where satClock is the correction for the satellite clock error computed using the information contained in the navigation message, and it includes the Time Group Delay (TGD) [27] and the relativistic errors Rel [2]

(3) $$satClock=clock_{bias}+TGD+Rel.$$

where $clock_{bias}$ is the satellite clock bias computed using a polynomial model whose parameters are provided in the satellite navigation message.

Sag is the correction for the Sagnac effect [28], and it is computed as described in [2]. If not corrected, the Sagnac effect can lead to an error up to 30 m [28]. *T* is the correction of the dry component of the tropospheric error obtained using the Hopfield model [29], and *I* is the ionospheric correction computed, in this case, using the NeQuick-G model [14].

The corrected measurements are then used for the PVT estimation using a Weighted Least Squares (WLS). The weights are based on the satellite elevation as described in [30]. Using this approach, the user position and the receiver clock bias, $cdt_{r}$, are computed. A WLS approach was used in order to avoid temporal smoothing effects that could have masked phenomena occurring on an epoch-by-epoch basis. These smoothing effects are typically introduced by Kalman Filter (KF)-based navigation solutions, which were not considered in this work. Note that the velocity component of the PVT solution is computed using Doppler measurements. Since velocity components were not analyzed in this paper, Doppler measurements and their usage are not described here.

### 2.2. RE Estimation

The RE is sum of the errors projected on the distance between the receiver and the satellite, and it includes errors due to the different elements impacting the position computation [31]: ephemeris error, atmospheric effects, multipath, receiver noise, etc. The RE represents the residual error experienced at the user level after the correction of all the possible error sources and includes the remaining errors due to the model imperfections. In the experiments conducted, the user position is known, and the geometric distance *d* in Equation (1) is computed from the satellite/receiver positions. The models adopted for the error corrections have been already presented. Thus, the only remaining term is the receiver clock bias, $cdt_{r}$, which has been estimated considering the known receiver location.

Thus, the RE is computed as

(4) $$RE=\rho_{c}-d+\tilde{cdt_{r}}$$

where $\tilde{cdt_{r}}$ is the estimated receiver clock bias.

In order to evaluate the impact at the receiver level of the proposed approach, the RE has been estimated; in particular, the difference between REs with different update intervals and the baseline RE computed updating the ionospheric corrections at each epoch has been evaluated.

(5) $$\Delta RE_{UI}\left(k\right)=RE_{UI}\left(k\right)-RE_{UI}\left(1\right).$$

In Equation (5), index UI denotes the update interval, whereas *k* is the epoch index. When UI>1, the ionospheric corrections are kept constant for UI seconds. The UI is the time between ionospheric correction updates. Thus, ionospheric corrections are kept constant for UI seconds. The minimum interval has been set to 1 s, which corresponds to the update rate of the position solution. The maximum interval considered in this work has been set to 2 h; this value is sufficiently large to have significant changes in the ionospheric conditions. The PVT solution and the related quantities, such as the RE, are updated each second.

## 3. NeQuick-G

Different algorithms for the computation of the ionospheric corrections are currently available; GPS adopts the Klobuchar model [13,17] while Galileo uses NeQuick-G [14]. The two models have different performances and have been compared in several works [20,32,33]. The comparison between Klochar and NeQuick-G is out of the scope of this work.

Given its complexity, reference software implementations of NeQuick-G have been made available to researchers and to receiver manufacturers. A first, NeQuick-G implementation in C was provided by European Space Agency (ESA) (https://essr.esa.int/project/nequickg-galileo-ionospheric-correction-model). A new optimized version of the NeQuick-G algorithm was developed by the European Commission Joint Research Centre (JRC). The implementation is released as a free and open source software under the terms of the European Union Public Licence (EUPL), version 1.2. The code of this NeQuick-G implementation is available at https://www.gsc-europa.eu/support-to-developers/nequick-g-source-code and has been used in this work for studying the impact of the correction update rate. Finally, a C++ implementation of the model was developed by the authors of [23].

Several studies focus on the performance of the NeQuick-G model. The authors of [34] evaluated the model during the Galileo In Orbit Validation (IOV) phase. The residual ionospheric Slant Total Electron Content (STEC) error, as defined in [14], was evaluated by [35] using the signals broadcast by the Galileo In Orbit Validation Element (GIOVE) satellites. Performance of the NeQuick-G model between March 2013 and December 2016 was discussed in [36]. The main limitation to the usage of the NeQuick-G model is its computational load, which mainly is due to the numerical integration needed to compute the STEC. In particular, height-dependent electron density profiles above numerous foot points have to be evaluated, leading to a large computational load [23]. The effort required for running NeQuick-G has been estimated to be 60 times larger than that needed for the Klobuchar model [24]. In order to reduce the computational burden, an alternative application of the NeQucik-G model is presented in the following section.

### A Smart Application of NeQuick-G

**Figure 1:**
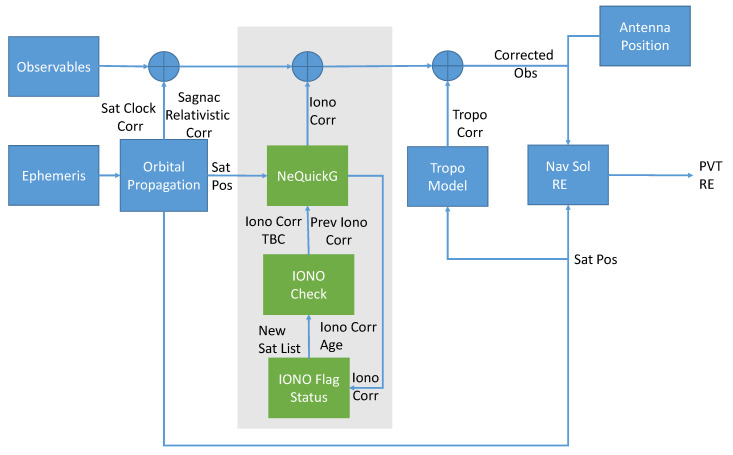
Schematic representation of the processing adopted for evaluating the impact of the update rate on computing the ionospheric correction.

Figure 1 provides a schematic representation of the processing adopted for the analysis considered in this paper. The observables obtained from a smartphone are corrected using models based on data provided by the signal navigation message (ephemeris block) and adopted for the computation of the user PVT and RE. The main focus of this research is on the functional blocks in the light grey box in Figure 1; these blocks are responsible for the computation of the ionospheric corrections. In the standard approach, ionospheric corrections are computed for all the satellites in view and for each epoch; hence, in a standard implementation, only the NeQuick-G block in the grey box would be present. In the proposed approach, the ionospheric corrections are computed only at specific epochs and kept constant for short periods of time. Thus, different update rates for the ionospheric corrections are adopted. In order to implement this approach, two additional elements have been introduced. Although these two additional blocks slightly increase the complexity of the navigation algorithm, they allow a reduction of the overall processing time by controlling the update of the ionospheric corrections. The additional elements are the “Iono Flag status” and the “Iono Check” blocks. The first one verifies if there are new satellites for which the ionospheric corrections need to be computed, and it associates to the ionospheric corrections an age. The ionospheric correction age is computed as the difference between the current epoch and the epoch at which the correction was computed. The ionospheric flag status is a preliminary check performed just before the PVT computation.

The ionospheric correction age and the list of the new satellites are then passed to the “Iono check” block, which identifies the ionospheric corrections to be computed. In particular, the corrections are computed for all the new satellites and for the satellite having corrections with an age larger than the update interval. In this research, 22 different update intervals have been considered from two seconds to two hours. The ionospheric corrections obtained using this approach and the NeQuick-G model were used to correct the pseudorange measurements, as shown in Equation (2).

## 4. Experimental Set-Up

In order to assess the impact of the update interval of the NeQuick-G correction computation, a dedicated experimental set-up was developed where a dual-frequency, multi-constellation Android device was kept static and used to collect several hours of Android raw measurements. An Android smartphone was placed inside a shielding box equipped with a wideband antenna [37]. The input of the shielding box was connected to a rooftop multi-frequency geodetic antenna. The antenna integrated within the shielding box was used to re-radiate GNSS signals inside the box. This type of set-up provides optimal visibility conditions for the data collection and allows one to keep the smartphone in a controlled lab environment. Moreover, the position of the rooftop antenna was carefully surveyed. Since the smartphone derived measurements related to the rooftop antenna, it was possible to assess position errors.

Finally, the smartphone was powered through the Universal Serial Bus (USB) connector integrated in the shielding box. Thus, it was possible to perform long data collections without interruptions. Almost 24 h of continuous data were collected.

Android raw measurements were converted in Receiver INdependent EXchange (RINEX) format and used for the analysis. The phone adopted for the data collection was a Xiaomi Mi 8.

**Figure 2:**
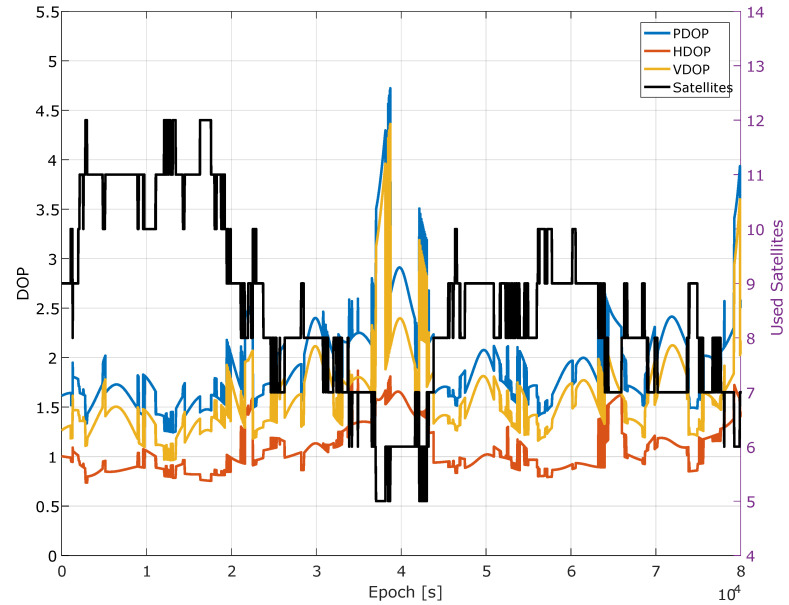
Dilution of Precision (DOP) values and number of satellites as a function of time.

Dedicated processing tools implementing the scheme detailed in Section 2 were developed and used for the analysis. Geometry conditions and number of satellites used in the different PVT solutions are analyzed in Figure 2. In this case, the analysis is performed as a function of time. From the figure, it emerges that almost 24 h of data were analyzed with a number of GPS satellites ranging from a minimum of 5 to a maximum of 12. As expected, higher Dilution Of Precision (DOP) values were obtained when a low number of satellites were used in the PVT solution.

The ionosphere was calm during the experiment, in particular the Ap index [38] had a variation between 12 and 22 with a daily mean of 16.

## 5. Experimental Analysis

### 5.1. Analysis Metrics

In order to evaluate the impact of the update rate of the NeQuick-G corrections, different metrics have been considered. The overall computation load was analyzed with focus on the following metrics:

- the overall number of corrections required for the processing of the whole data-set;
- the execution time as measured through Matlab primitives.

The number of corrections is a function of the correction update rate, of the experiment duration, and of the satellite availability.

The execution time, as measured through Matlab primitives, quantifies the time required to process the full data-set and includes the computational requirements for the evaluation of the navigation solution. In this respect, this metric provides an indication of the computational cost of NeQuick-G relative to the computation of the navigation solution. The reduction of the update rate significantly alleviates the computational requirements of the ionospheric corrections.

For the evaluation of the execution time, the data-set introduced in Section 4 was processed 10 times for each update interval. For each run, the processing time was measured, and the final execution time was estimated as the median of these 10 realizations of the processing time. In order to limit the effect of additional parallel processes, a dedicated Personal Computer (PC), running Matlab software, was used for the processing. In this way, random effects related to factors external to the processing of the data-set were limited.

In addition to the computational load, the impact of an increased update interval was analyzed in terms of ionospheric correction changes, RE, and final position solution differences. When a lower update rate is used, older ionospheric corrections are adopted for the pseudorange adjustment. The analysis in terms of ionospheric correction changes quantifies the differences between corrections obtained with different update rates. The RE defined in Section 2.2 quantifies the overall impact of the update rate on the corrected ranges. Finally, the changes in the position solution are used to identify potential degradations caused by lower update rates for the ionospheric corrections.

### 5.2. Computational Load Results

**Figure 3:**
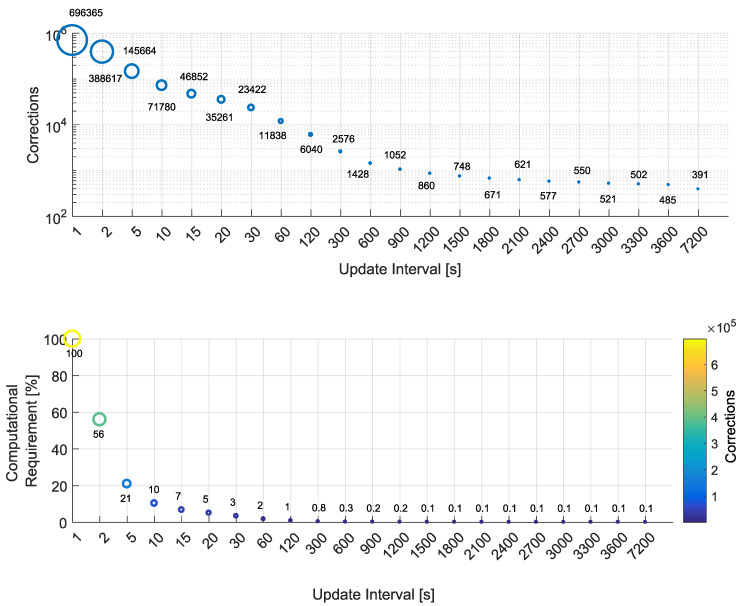
Upper part: Number of ionospheric corrections as a function of the update interval. Lower part: Computational requirements as a function of the update interval. An update interval equal to 1 is used as reference.

Results in terms of computational load are presented first. In particular, the upper part of Figure 3 shows the number of corrections computed as a function of the update interval. For an update interval equal to 1 s, which represents the baseline condition, about $7\times 10^{5}$ corrections are computed. This value is determined by the duration of the data-set (about a day) and by the satellite availability (on average, 8 satellites were present). For visualization purposes, the vertical axis of the upper part of Figure 3 is in logarithmic scale, while all the other axes adopt a linear scale.

When the update interval is increased to 2 s, the number of corrections almost halves. Note that a reduction of 50% was not achieved since the system has to handle satellite acquisitions and re-acquisitions. When a satellite is acquired/re-acquired, its ionospheric corrections are computed independently from the update rate. Figure 2 shows that frequent changes in the number of satellites occurred. These changes force the computation of ionospheric corrections that prevents the 50% reduction expected for an update interval equal to 2 s.

In general, the number of corrections decreases with the increase of the update interval; however, the number of corrections is not halved when the update rate is doubled for the presence of satellite acquisitions and re-acquisitions. In the bottom part of Figure 3, the computational requirement as a function of the update interval is shown. The computational requirement is computed as

(6) $$ComReq=100-\left(100\cdot \left(\frac{NumCorr_{UI}\left(k\right)-NumCorr_{UI}\left(1\right)}{NumCorr_{UI}\left(1\right)}\right)\right)$$

where $NumCorr_{UI}\left(k\right)$ is the number of ionospheric corrections computed using an update interval of *k* seconds.

When the update interval is increased to 30 s, the reduction in the number of corrections is equal to 97%, and the computational requirement is 3% of that of the baseline case. When the update interval is further increased to 600 s, a reduction close to 100% is achieved. In this case, the number of residual corrections (2576) is about 0.3% of the initial number of corrections. The computational requirements converge toward the values of 0.1 with update intervals larger than 1500 s. Further increasing the update interval does not provide significant reductions since a minimum number of corrections is required to handle satellite signal acquisition and re-acquisitions.

These results show that even small increases of the update interval provide significant reductions in terms of number of corrections and associated computational requirement.

**Figure 4:**
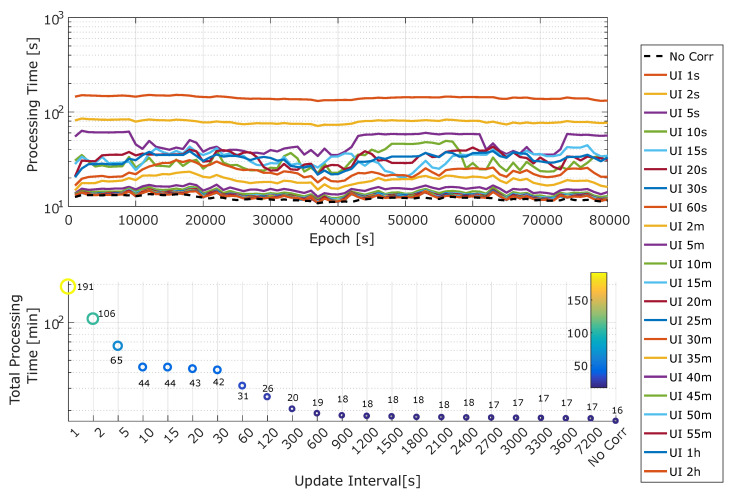
Upper: Median processing time required to process 1000 epochs as a function of time and of the update interval. Lower: Median total processing time as a function of the update interval. In the legend, “UI” stands for “Update Interval”.

The processing time evaluated for different update intervals is analyzed in Figure 4. The upper part of the figure shows the average time required for computing 1000 epochs, the vertical axis is in logarithmic scale. For the baseline case, about 150 s is required. This time requirement is almost halved when moving to an update interval equal to 2 s. No significant improvements are observed when the update rate is above 5 min. This is due to the fact that the execution time evaluated in the upper part of Figure 4 also accounts for the computation of the navigation solution.

The dashed line in the upper part of Figure 4 represents the time required to compute the navigation solution without ionospheric corrections. As the update interval increases, the processing time progressively approaches this line, which can be considered as a lower bound for this metric. For update intervals on the order 5 min, the processing time practically coincides with the lower bound obtained in the absence of corrections.

The lower part of Figure 4 shows the overall time required to process the full data-set. The figure confirms that a significant processing time reduction was obtained and reduced the update rate. The most notable processing time improvement was obtained when moving to an update interval equal to 10 s. In this case, the processing time is reduced by a factor greater than 4.3. For larger update intervals, a more limited improvement is obtained. In particular, for update intervals greater than 5 min (300 s), only marginal improvements were achieved.

### 5.3. Effects on Ionospheric Corrections

The effect of the update rate on the ionospheric corrections has been evaluated by considering ionospheric correction differences:

(7) $$\Delta I_{UI}\left(k\right)=I_{UI}\left(k\right)-I_{1}\left(k\right)$$

where UI is the update interval, $I_{UI}\left(k\right)$ is the *k*th ionospheric correction calculated with an update interval equal to UI, and $I_{1}\left(k\right)$ is the *k*th baseline ionospheric correction calculated with an update interval equal to 1 s. For UI>1, $I_{UI}\left(k\right)$ is kept constant for UI epochs. Ionospheric correction differences allow one to determine the effect of the update rate on the corrections.

**Figure 5:**
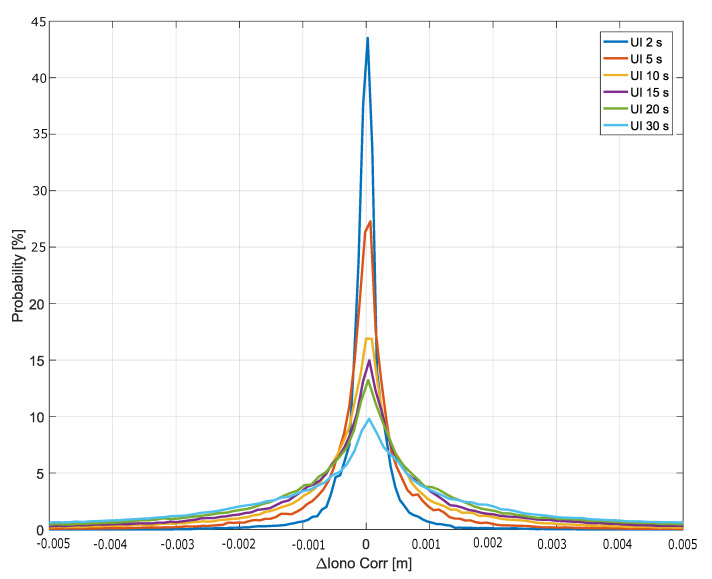
Empirical histograms of the ionospheric correction differences for different update intervals. Histograms have been represented with continuous lines to allow the superposition of different curves. In the legend, “UI” stands for “Update Interval”. The histograms have been computed considering epochs without updated corrections.

The probability density functions (pdfs) (empirical histograms) of the ionospheric correction differences are shown in Figure 5 for update intervals from 2 to 30 s. The histograms have been computed considering only epochs without updates. For example, when an update interval equal to 2 s is considered, for approximately 50% of the time, $I_{2}\left(k\right)$ coincides with $I_{1}\left(k\right)$. This condition happens when an update occurs. These epochs with an update have been removed in order to avoid to bias the pdfs and inflate probability masses around zero. Despite this fact, all the histograms of $\Delta I_{UR}\left(k\right)$ are centered around zero. Probability masses around zero decrease with the increase of the update interval, while for larger update intervals more spread histograms are obtained; these ionospheric correction differences are well within 1 cm of magnitude.

This shows that increasing the update interval of the NeQuick-G corrections to 30 s does not significantly bias the ionospheric corrections with differences in the cm level.

**Figure 6:**
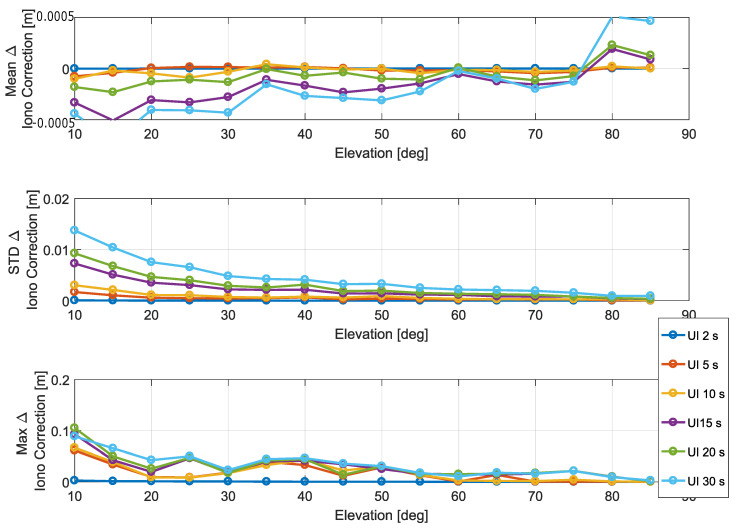
Statistical parameters of the ionospheric correction differences as a function of the elevation angle. In the legend, “UI” stands for “Update Interval”.

Statistical parameters of the ionospheric correction differences are analyzed in Figure 6. Mean, standard deviation, and maximum absolute values of the difference of the ionospheric corrections are provided as a function of the elevation angle. For elevation angles between 35 and 75 degrees, the mean of the correction differences was practically negligible, with values in the order of 0.1 mm. While higher values were observed for high/low elevation angles, mean correction differences did not exceed 0.5 mm. For the mean value, a clear dependence on the elevation angle cannot be established since, for all the elevation angles, differences are in the sub-millimeter order.

The standard deviation of the ionospheric correction differences decreases with the elevation angle and increases with the update interval. In the worst case, corresponding to an update interval equal to 30 s, a standard deviation of 1.5 cm is obtained.

The maxima of the absolute correction differences are shown in the bottom part of Figure 6 with values around 10 cm. These maximum values are obtained for an update interval equal to 30 s.

**Figure 7:**
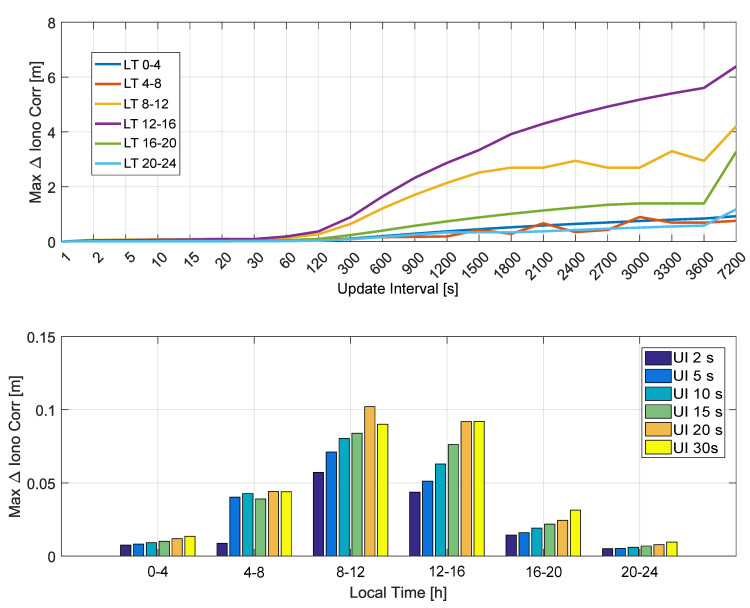
Maximum absolute ionospheric correction differences as a function of the local time. In the legend, “UI” stands for “Update Interval”.

Finally, the maximum absolute ionospheric correction differences are analyzed as a function of the local time in Figure 7.

In the upper part of Figure 7, maximum absolute ionospheric correction differences are computed by considering time slots of 4 h. Each slot leads to a curve that is a function of the update interval. These curves are analyzed in the upper part of Figure 7 and show that ionospheric correction differences are negligible for update intervals lower than 60 s. Moreover, the curves show that ionospheric correction differences are higher for the (8–12) and (12–16) time intervals.

These results are confirmed by the bottom part of Figure 7 that better analyzes the ionospheric correction differences for update intervals between 2 and 30 s. While the (8–12) and (12–16) time intervals are those more affected by the update rate, the differences are again within 10 cm. The dependence on the local time is expected since, in the (8–16) local time range, ionosphere is more active and thus more variable over time. While the NeQuick-G model is able to follow these variations, an increased update interval reduces this ability, and higher differences are observed. These differences are, however, in the centimeter range for update intervals up to 30 s.

### 5.4. Effects on Ranging Error

**Figure 8:**
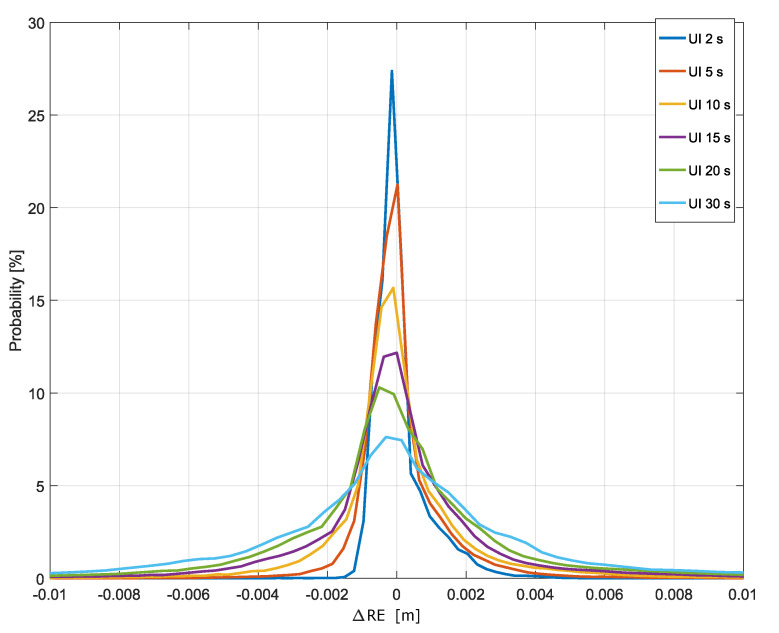
Empirical histograms of the RE differences for different update rates. Histograms have been represented with continuous lines to allow the superposition of different curves. In the legend, “UI” stands for “Update Interval”. The histograms have been computed considering epochs without updated corrections.

The effect of the update rate on the RE is analyzed in this section. The analysis is based on the RE differences defined in Equation (5), and it aims at quantifying the final effect on the pseudoranges. Figure 8 shows the empirical histograms of the RE differences obtained for different update intervals. Note that RE differences capture different effects on the corrections of pseudoranges and differ from ionospheric correction differences that do exploit information such as the user position. In this case, the histograms have also been computed using only epochs without correction updates. Despite this fact, a clear probability peak was found around zero. As for the ionospheric correction differences, the probability masses around zero decreased with the increase of the update interval. Nevertheless, most of the RE differences had magnitudes lower than 1 cm. In the worst case, obtained for an update interval equal to 30 s, only 15% of the RE differences had a magnitude greater than 1 cm, whereas the number of RE differences greater than 10 cm could be neglected (0.004%).

**Figure 9:**
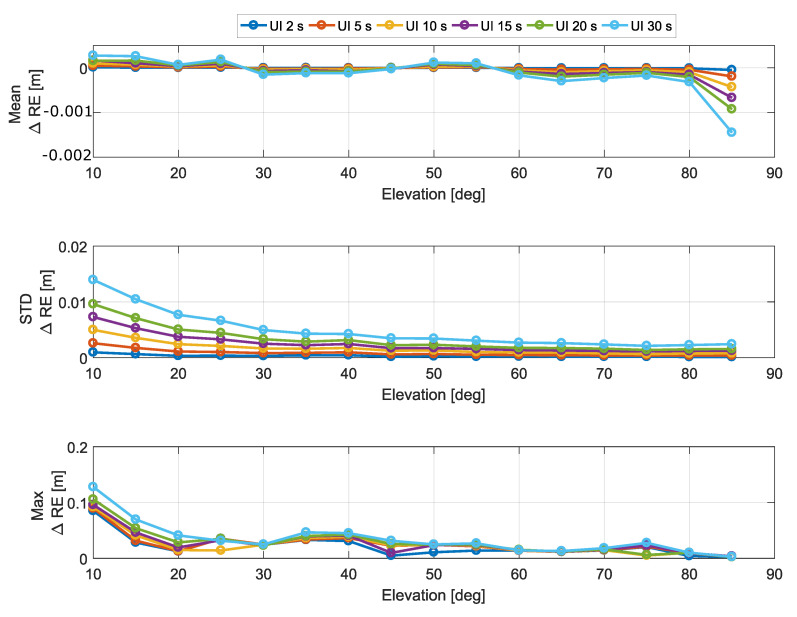
Statistical parameters of the RE differences as a function of the elevation angle. In the legend, “UI” stands for “Update Interval”.

Statistical parameters for the RE differences are analyzed in Figure 9 as a function of the elevation angle.

As for the ionospheric correction differences analyzed in Section 5.3, the mean RE differences are in the sub-millimeter range. The largest deviations were observed for elevation angles close to zenith (90 degrees) with values up to 1.5 mm in magnitude. These values are well below the magnitude of most errors affecting pseudoranges.

The standard deviation of the RE differences is analyzed in the middle box of Figure 9. The standard deviation decreased with the elevation angle and increased with the update interval. In the worst case, obtained for an update interval equal to 30 s and for a 10 degree elevation angle, a standard deviation of about 1.5 cm was observed. These values are in the same range of the standard deviations observed for the ionospheric correction differences.

Finally, the maximum absolute RE differences are considered in the bottom part of Figure 9. The maximum deviation observed was lower than 13 cm. These results indicate that update intervals up to 30 s have a negligible impact on the final RE.

**Figure 10:**
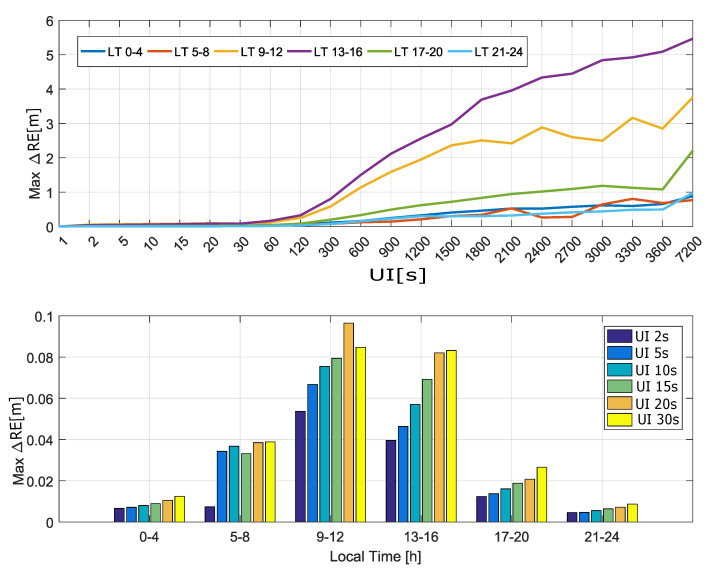
Maximum absolute RE differences as a function of the local time. In the legend, “UI” stands for “Update Interval”.

Finally, RE differences are analyzed in Figure 10 as a function of the local time. As for the ionospheric correction differences, the local time was divided in six time slots, each of 4 h. For each time slot, the maximum absolute RE differences were computed, and a curve was derived as a function of the update interval. These curves are provided in the upper part of Figure 10. While the largest deviations were observed in the (8–12) and (12–16) time slots, the maximum absolute RE differences were below 1 m for update intervals up to 300 s.

The maximum absolute RE differences are further analyzed in the bottom part of Figure 10 for update intervals up to 30 s. The results confirm the limited impact of increasing the update interval.

### 5.5. Effects on the Final Position Estimation

The effects on the position solution are finally analyzed in this section. In particular, position solution errors have been first computed. The position solution is expressed in a local East North Up (ENU) frame centered on the reference position of the rooftop antenna connected to the shielding box; hence, the obtained coordinates represent the East, North, and Up error components, respectively. For clarity, the following notation has been adopted: $e_{UI,i}\left(k\right)$ is the *i*th component of the position error computed at the *k*th epoch with an update interval equal to UI, with the index i=e,n,u.

The horizontal position error has been evaluated as

(8) $$e_{UI,h}\left(k\right)=\sqrt{e_{UI,e}^{2}\left(k\right)+e_{UI,n}^{2}\left(k\right)}.$$

Similarly, the vertical position error was obtained as

(9) $$e_{UI,v}\left(k\right)=\left|e_{UI,u}\left(k\right)\right|.$$

Finally, error differences have been computed as

(10) $$\begin{array}{c} \Delta e_{UI,h}\left(k\right)=e_{UI,h}\left(k\right)-e_{1,h}\left(k\right)\\ \Delta e_{UI,v}\left(k\right)=e_{UI,v}\left(k\right)-e_{1,v}\left(k\right) \end{array}$$

where $e_{1,h}\left(k\right)$ and $e_{1,v}\left(k\right)$ are the position errors obtained using the baseline configuration with an update interval equal to 1 s. Position error differences quantify potential degradations of the position solution accuracy. A negative difference indicates that the position error was actually reduced.

**Figure 11:**
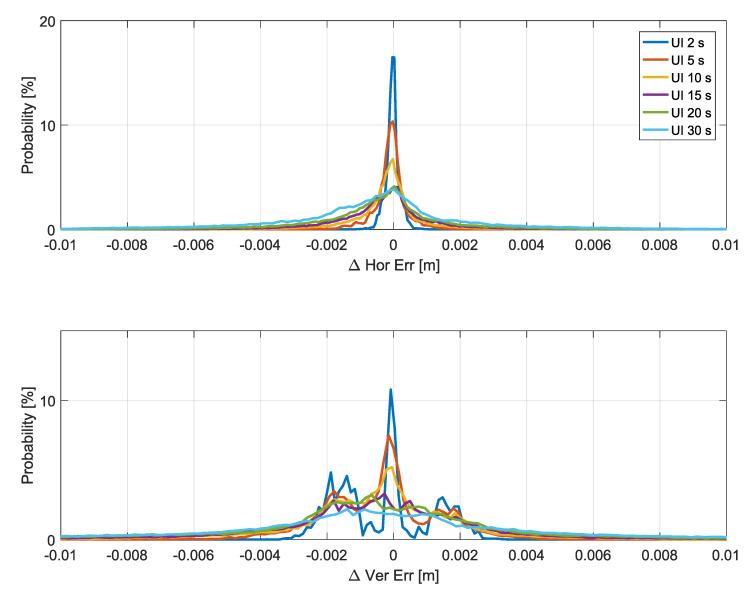
Empirical histograms of the position error differences for different update intervals. Upper: Horizontal position error differences. Lower: Vertical position error differences. Histograms have been represented with continuous lines to allow the superposition of different curves. In the legend, “UI” stands for “Update Interval”. The histograms have been computed considering epochs without updated corrections.

The histograms of the position error differences defined in Equation (10) are shown in Figure 11. As for the pdfs analyzed in the previous sections, only epochs when an update is not performed are considered.

**Figure 12:**
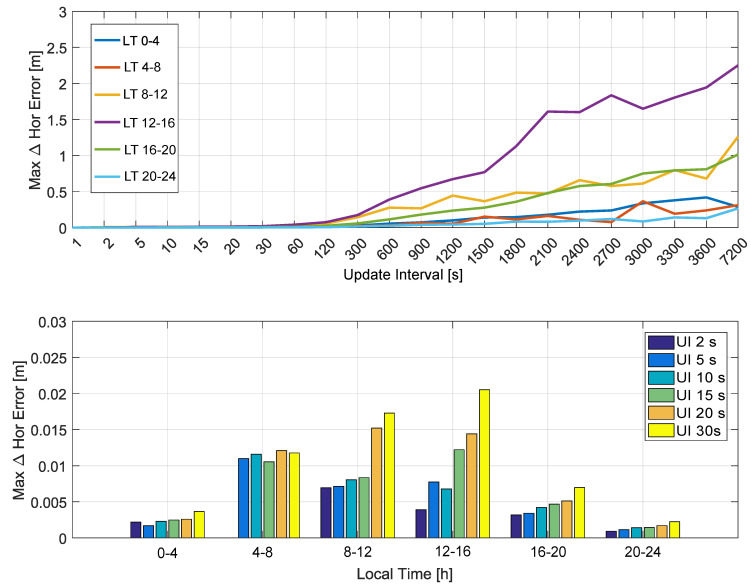
Maximum absolute horizontal position differences as a function of the local time. In the legend, “UI” stands for “Update Interval”.

**Figure 13:**
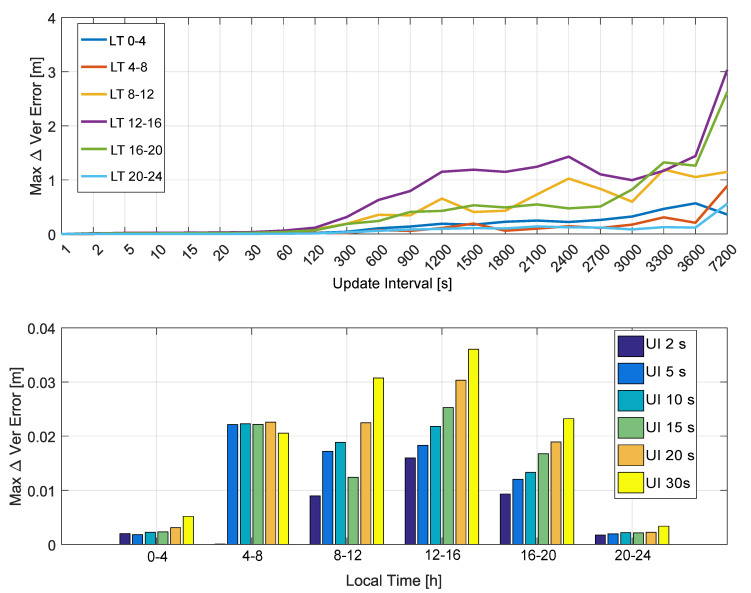
Maximum absolute vertical position differences as a function of the local time. In the legend, “UI” stands for “Update Interval”.

The histograms show that both horizontal and vertical position error differences were below 1 cm. In both horizontal and vertical cases, the histograms were practically symmetric, indicating that the variations caused by larger update intervals are random in nature and can cause either a reduction or an increase of the position errors. While larger variations were observed for the vertical case, these differences can be neglected with respect to the overall error budget of a SPP solution. As for the other metrics analyzed in the paper, the histograms become more spread as the update interval increases. The maximum position error differences are analyzed in Figure 12 and Figure 13, which consider the horizontal and vertical components, respectively. The analysis is provided as a function of the local time. Decimeter-level deviations were observed for update intervals up to 300 s for all the time slots considered. Similarly to the other metrics analyzed, the (12–16) interval was the one leading to the largest deviations. These differences were, however, limited to about 10 cm if update intervals lower than 300 s were considered. Update intervals up to 30 s are analyzed in the bottom part of Figure 12 and Figure 13. The figures confirm that very limited position error differences were obtained for update intervals up to 30 s. For the horizontal channel, the deviations were limited to about 2 cm, whereas for the vertical case the maximum differences were lower than 4 cm.

These results confirm that the update interval of the NeQuick-G corrections can be safely increased to 30 s without compromising the quality of the navigation solution.

## 6. Conclusions

In this paper, a smart application of the NeQuick-G algorithm has been investigated. The approach is based on the assumption that ionospheric errors can be considered constant over short time intervals. Thus, the update rate of the ionospheric corrections can be significantly decreased, with a significant reduction of the computational burden. An ad hoc navigation software was developed to integrate the proposed approach into the navigation solution.

The developed navigation algorithm was tested using real data collected by a static smartphone. In order to evaluate the reduction of the computational burden using an update interval larger than one second, the processing time was measured. The paper demonstrates that significant processing time reduction can be achieved by increasing the update interval of the NeQuick-G corrections. Increasing the update interval to 10 s provides a 4.3 times reduction of the processing time with respect to the baseline solution with a 1 Hz update rate.

In order to assess the degradation caused by an increased update interval of the ionospheric corrections, several metrics were analyzed in the ionospheric correction, measurement, and position domains.

In the ionospheric correction domain, which was analyzed in Section 5.3, negligible impacts with differences on the milliliter order have been observed for update intervals up to 30 s.

In the position domain, considered in Section 5.5, position differences below 1 m were observed with update intervals in the order of 5 min.

The developed approach is particularly suited for devices with limited computational resources, such as Android smartphones. This allows one to reduce the computational load of the NeQuick-G algorithm. The update interval can be selected according to the application requirements.

Finally, a long data collection was used to analyze the impact of the local time on the magnitude of the ionospheric correction differences. From the analysis, it emerged that the time slot between 8 and 16 (local time) is the most affected by the update rate. Between 8 and 16 h local time, ionosphere is more active and thus more variable over time. While the NeQuick-G is able to follow these variations, an increased update interval reduces this ability, and higher differences are observed. These differences are, however, in the centimeter range for update intervals up to 30 s. With this in mind, it is also possible to vary the update interval of the ionospheric corrections as a function of the local time; update intervals up to 120 s could be considered outside the (8–16) time slot.
